# The role of galectin-3 in atrial fibrillation

**DOI:** 10.1007/s00109-023-02378-5

**Published:** 2023-09-29

**Authors:** Grzegorz Procyk, Aleksandra Czapla, Kamila Jałocha, Agata Tymińska, Marcin Grabowski, Aleksandra Gąsecka

**Affiliations:** 1st Chair and Department of Cardiology, Medical University of Warsaw, Banacha 1A, 02-097 Warsaw, Poland

**Keywords:** Galectin-3, Atrial fibrillation, Biomarker, Fibrosis

## Abstract

Numerous risk factors for atrial fibrillation (AF) progression have been identified. However, the biomarkers mentioned in the guidelines do not have any clinically relevant predictive value. Some research groups investigated the potential utility of galectin-3 (gal-3) as a diagnostic, prognostic, and predictive biomarker in AF. In this review, we have thoroughly summarized the current data on the role of gal-3 in AF based on the original research in this field. Patients suffering from AF present with increased levels of gal-3. The concentration of gal-3 differs between patients with AF depending on the type of AF — it is higher in patients with persistent AF than in patients with paroxysmal AF. Multiple studies investigating the reappearance of AF in patients who underwent ablation have shown that gal-3 is a promising biomarker to predict the outcome of this therapy. Patients with increased levels of gal-3 are at higher risk of AF recurrence. Although the research considered in this work addressed many aspects of the role of gal-3 in AF, most of it has been conducted on a small group of patients. Therefore, further research and extensive clinical trials confirming described findings are highly warranted.

## Introduction

According to the European Society of Cardiology (ESC) definition, atrial fibrillation (AF) is a supraventricular tachyarrhythmia with uncoordinated atrial electrical activation and consequently ineffective atrial contraction [1]. More than 60% of patients suffering from AF have a decreased quality of life. AF is a significant risk factor for ischaemic stroke and heart failure, increasing mortality [2]. Currently, the estimated prevalence of AF in adults is between 2 and 4%, being the most frequent cardiac arrhythmia nowadays [1, 3]. AF incidence and prevalence have increased over the last 20 years and will likely continue rising over the next 30 years [3].

AF progression from paroxysmal to non-paroxysmal is associated with multiple risk factors, including age, and many diseases, i.e., heart failure, hypertension, chronic kidney disease, chronic pulmonary diseases, diabetes mellitus, and previous stroke [1, 4]. However, the biomarkers mentioned in the guidelines do not have clinically relevant predictive value [1]. Because of that, further research on the role of biomarkers in AF is highly warranted [5, 6]. Knowledge about the pathogenesis of the disease should always be the rationale for the search for potential biomarkers. The ongoing fibrosis in the left atrium is one of the leading causes of AF since it delays electromechanical conduction and creates a substrate for AF [7]. Multiple proteins involved in fibrosis have been investigated so far. Recently, much scientific attention has been drawn to galectin-3 (gal-3).

Gal-3 is a β-galactoside-binding protein belonging to the lectin family. It plays a vital role in many physiological cellular functions, including cellular growth, differentiation, proliferation, apoptosis, cellular adhesion, and tissue repair [8]. The concentration of gal-3 levels can be measured with the use of immunoassays. Enzyme-linked immunosorbent assay (ELISA) is the most commonly used technique. Nevertheless, other variants of immunoassays can also be used, including enzyme-linked fluorescent assay (ELFA) or chemiluminescent microparticle immunoassay (CMIA). Although there are some differences in the specificity and sensitivity of these tests, price, and availability seem to be decisive factors for test choice, favoring ELISA.

How gal-3 is implicated in the pathogenesis of various cardiovascular diseases has been extensively explored, mainly referring to its involvement in inflammation and tissue fibrosis processes [8, 9]. Much research has focused on its role in AF.

Some research groups investigated the potential utility of gal-3 as a diagnostic and disease severity/prognostic biomarker in AF. In contrast, others studied the correlation between gal-3 concentrations and the possibility of AF recurrence after different interventions, such as ablation [10, 11]. The understanding of the role of gal-3 in the induction and the progression of AF may lead to better management of patients as well as may provide new targets for treatment.

In this review, we have thoroughly summarized the current data on the role of gal-3 in AF based on the original research in this field. We aim to contribute to setting objectives for future research on gal-3 and its use as a biomarker in daily clinical practice.

## Galectin-3 as a potential biomarker in atrial fibrillation

**Fig. 1:**
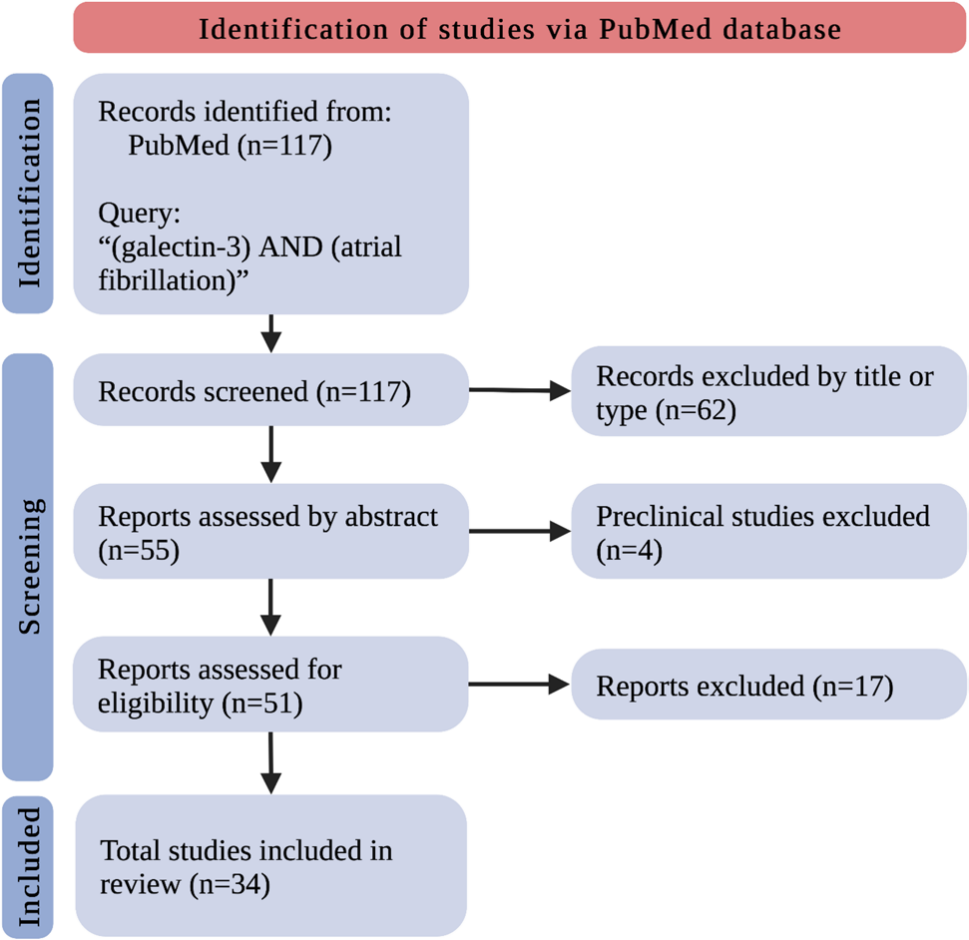
The flowchart for the selection process; *n*—a number of studies

Numerous clinical studies investigating the role of gal-3 in AF have already been conducted. In our review, we included only original clinical research. Reviews, letters to the editors, and commentaries were not included. We searched PubMed Database by the query: “(galectin-3) AND (atrial fibrillation)” which yielded a total of 117 records. Excluding unsuitable titles, article types, or abstracts, we retrieved in complete form and assessed 51 studies. We evaluated the complete data reports for eligibility and excluded 17 studies irrelevant to the field. We eventually included 34 original clinical research relevant to the discussed area (Fig. 1).

**Fig. 2:**
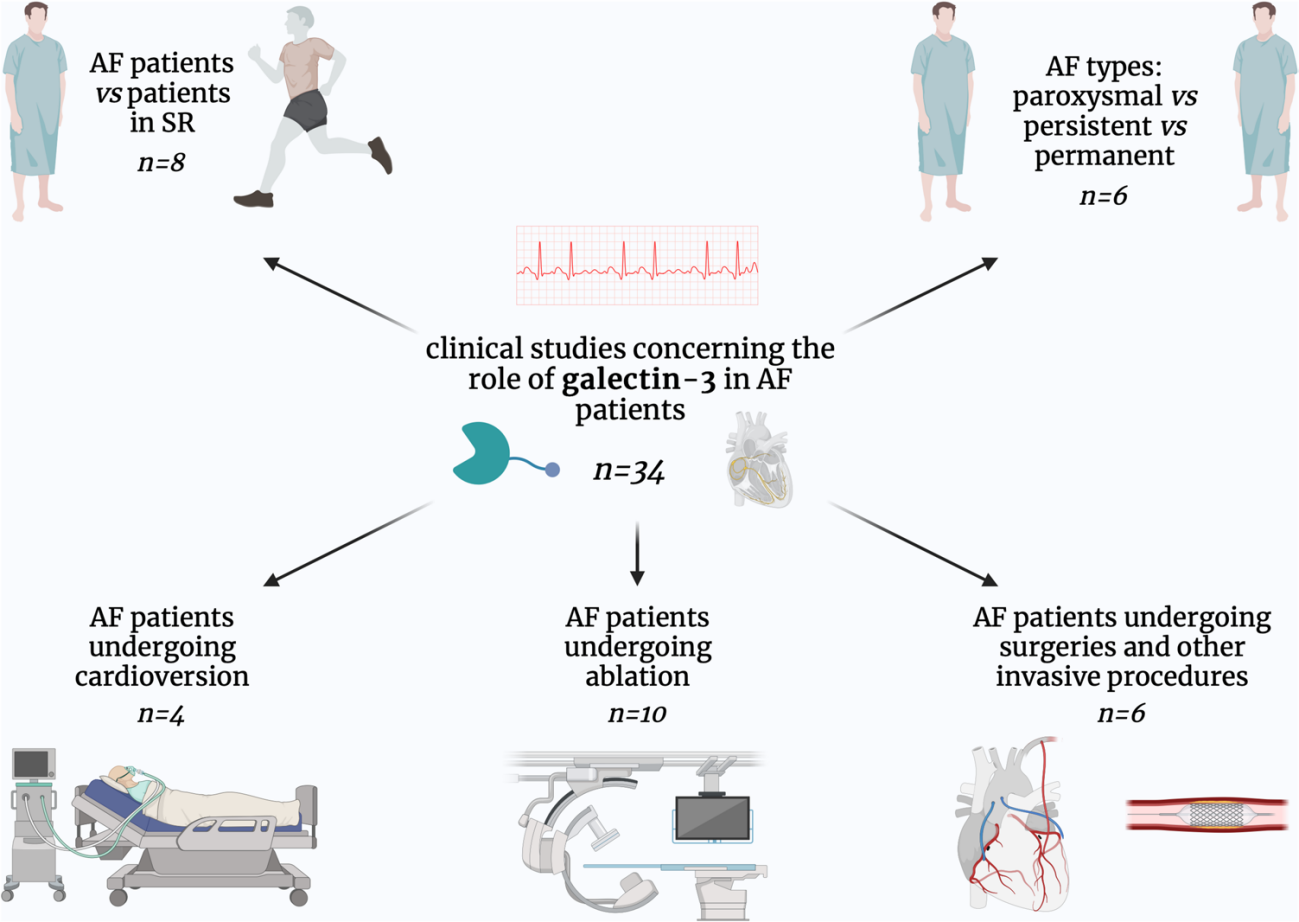
Graphical presentation of clinical studies included in this review, divided into subtopics; AF—atrial fibrillation; *n*—a number of included clinical studies in a given field; SR—sinus rhythm

We divided these studies into the following parts: (i) differences in the levels of gal-3 between patients with AF and patients in sinus rhythm, (ii) differences in the levels of gal-3 between patients with different types of AF, (iii) gal-3 in patients with AF undergoing cardioversion, (iv) gal-3 as a predictor of AF recurrence after ablation and (v) gal-3 in patients with AF undergoing surgeries and other invasive procedures (Fig. 2).

### Differences in the levels of galectin-3 between patients with atrial fibrillation and patients in sinus rhythm

Ho et al. studied the relationship between gal-3 levels and the probability of AF occurrence in participants of the Framingham Offspring cohort. They discovered that higher concentrations of circulating gal-3 were associated with an increased risk of developing AF [12]. Another research group compared the levels of gal-3 between patients with non-valvular AF and age-matched patients in sinus rhythm. It was shown that patients with AF had significantly higher concentrations of this biomarker than the control group. Moreover, the authors found a positive correlation between the concentration of gal-3 in blood and the left atrial volume index [13]. Consistently, Pauklin et al. analyzed the levels of gal-3 in patients suffering from paroxysmal or persistent AF admitted for electrical cardioversion or pulmonary vein isolation (PVI) and compared them with healthy controls. The study revealed that the levels of gal-3 were higher in patients with AF [14].

Similarly, Selcoki et al. analyzed gal-3 levels in patients with paroxysmal AF and preserved left ventricular systolic function and compared them with healthy controls matched by age and gender. It was shown that patients with AF had higher levels of gal-3 than controls. Moreover, the authors found a positive correlation between left atrial diameter and serum gal-3 levels [15].

Contrary results to these studies were found by Begg et al. They considered gal-3 level a marker of fibrosis in patients who underwent ablation. Therefore, they analyzed patients suffering from AF (paroxysmal or non-paroxysmal) who underwent ablation and compared them to non-AF control patients. They proved no significant difference in gal-3 levels between these groups [16]. However, the authors found that peripheral gal-3 levels were significantly higher than left atrial gal-3 levels.

Fashanu et al. studied the relationship between gal-3 levels and the probability of an AF incident. It was shown that patients with gal-3 levels ≥ 90th percentile had a higher risk of an AF incident than those with gal-3 levels < 90th percentile [17].

In another study, Shen et al. compared two groups of patients who underwent single mitral valve repair/replacement. The first group consisted of patients suffering from persistent AF, while the second group had sinus rhythm patients. It was proven that the levels of gal-3 measured in serum collected from coronary sinus blood and gal-3 measured in atrial tissues were higher in the first group than in the second one [18].

Hernández-Romero et al. analyzed gal-3 levels in patients with permanent AF who underwent cardiac surgery and compared them to those with aortic valve or ischaemic heart diseases who also underwent cardiac surgery. They observed differences in serum concentrations of gal-3 between the groups– gal-3 levels were higher in patients with permanent AF. Moreover, high levels of gal-3 were found to be an independent predictor of fibrosis [19]. All studies discussed in this section with additional information have been presented in Table 1.

**Table 1:** Summary of recent research investigating differences in the levels of galectin-3 between patients with atrial fibrillation and patients in sinus rhythm

**Ref.**	**Year**	**Population**	**Comparison**	**Outcome**	**Methods**
[12]	2014	3306 participants of the Framingham Offspring cohort	participants divided by the risk of developing AF	during a median follow-up of 10 years, 250 participants developed incident AF ↑ gal-3 lvl associated with an increased risk of developing AF	gal-3 measured in plasma by ELISA
[13]	2014	52 non-valvular AF pts	33 age-matched pts in SR	↑ gal-3 lvl in AF pts compared to pts in SR	gal-3 measured in serum by ELISA
[15]	2016	46 paroxysmal AF pts with preserved LV systolic function	38 age- and gender-matched control subjects	↑ gal-3 lvl in paroxysmal AF pts compared to controls ↑ LAD in paroxysmal AF pts compared to controls LAD correlated with gal-3 lvl in paroxysmal AF pts	gal-3 measured in serum by ELISA
[16]	2017	93 AF pts undergoing ablation (63 paroxysmal + 30 non-paroxysmal)	36 control patients	no significant difference in gal-3 lvl between the groups ↑ peripheral gal-3 lvl compared to left atrial gal-3 lvl	gal-3 measured in serum by ELISA
[17]	2017	8436 AF-free participants	pts divided by AF incident risk	during a median follow-up of 15.7 years, 1185 incident cases of AF were observed pts with gal-3 lvl ≥ 90^th^ percentile at higher risk of an AF incident compared to pts with gal-3 lvl < 90^th^ percentile	gal-3 measured in serum by CMIA technology
[19]	2017	15 permanent AF pts undergoing cardiac surgery	42 aortic valve disease pts + 58 IHD pts undergoing cardiac surgery	↑ gal-3 lvl in pts with permanent AF compared to aortic valve disease/IHD pts	gal-3 measured in serum by ELFA
[18]	2018	28 persistent AF pts undergoing single mitral valve repair/replacement	16 pts in SR undergoing single mitral valve repair/replacement	↑ gal-3 lvl in serum in AF pts compared to SR pts ↑ gal-3 lvl in atrial tissues in AF pts compared to SR pts	gal-3 measured in CS serum by ELISA gal-3 measured in atrial myocardium by WB and immunohistochemistry
[14]	2022	75 paroxysmal or persistent AF pts undergoing EEC or PVI	75 healthy age-, sex-, and blood pressure-matched individuals	↑ gal-3 lvl in AF pts compared to healthy individuals	gal-3 measured by CMIA technology

↑ increased, *AF* atrial fibrillation, *CMIA* chemiluminescent microparticle immunoassay, *CS* coronary sinus, *EEC* electrical cardioversion, *ELFA* Enzyme-Linked Fluorescent Assay, *ELISA* enzyme-linked immunosorbent assay, *gal-3* galectin-3, *IHD* ischaemic heart disease, *LAD* left atrial diameter, *LV* left ventricular, lvl levels,*pts* patients, *PVI* pulmonary vein isolation, *ref.* reference, *SR* sinus rhythm, *WB* western blotting

### Differences in the levels of galectin-3 between patients with different types of atrial fibrillation

Gurses et al. analyzed gal-3 levels in persistent or paroxysmal AF patients with preserved left ventricular systolic function and compared them to the age-and-gender-matched controls. It was proved that gal-3 levels and left atrial volume index were higher in AF patients than in the controls. Moreover, gal-3 levels were significantly higher in patients with persistent AF than in those with paroxysmal AF [20]. Accordingly, Chen et al. compared gal-3 levels between new-onset AF patients and patients with chronic AF. The study revealed that patients with new-onset AF had higher gal-3 levels than those with pre-existing, chronic AF [21].

Tang et al. compared patients with all three types of AF: paroxysmal, persistent, and permanent. It was shown that patients with persistent or permanent AF had higher levels of gal-3 compared to patients with paroxysmal AF. Moreover, gal-3 was an independent determinant of LAA thrombus in AF patients [22].

Another interesting research was held by Arbault-Biton et al. They divided the group of AF patients based on the AF duration: there were 47 patients with AF ≤ 48 h and 51 patients with AF > 48 h. It was proved that gal-3 concentrations did not differ between the groups [23].

Interestingly, Zaslavskaya et al. compared gal-3 levels in patients suffering from metabolic syndrome (MS) with coexisting paroxysmal/persistent AF or without arrhythmia to healthy controls. They showed that gal-3 levels were higher in patients with coexisting MS and AF than in patients with only MS and healthy controls. Moreover, gal-3 levels were higher in patients with persistent AF than in those with paroxysmal AF [24].

Wang et al. studied patients suffering from paroxysmal AF, dividing them based on the progression to persistent AF. It was demonstrated that patients with AF progression to a persistent state had higher gal-3 levels than those without AF progression. Moreover, gal-3 concentration was associated with AF progression [25]. All studies discussed in this section with additional information have been presented in Table 2.

**Table 2:** Summary of recent research investigating differences in the levels of galectin-3 between patients with different types of atrial fibrillation

**Ref.**	**Year**	**Population**	**Comparison**	**Outcome**	**Methods**
[20]	2015	76 paroxysmal or persistent AF pts with preserved LV systolic function	75 age-and-gender-matched controls	↑ gal-3 lvl and ↑ LAVI in AF pts compared to controls ↑ gal-3 lvl in persistent compared to paroxysmal AF pts	gal-3 measured in serum by ELISA
[21]	2016	32 NOAF pts	99 chronic AF pts	↑ gal-3 lvl in NOAF pts compared to chronic AF pts	gal-3 measured in plasma by ELISA
[24]	2016	50 MS with paroxysmal or persistent AF pts	50 MS pts w/o AF 50 healthy controls	↑ gal-3 lvl in MS AF pts compared to MS pts w/o AF and to healthy controls ↑ gal-3 lvl in persistent AF pts compared to paroxysmal AF pts	gal-3 measured in serum by ELISA
[22]	2019	153 nonvalvular AF pts	pts divided by AF type: 58 paroxysmal, 55 persistent, 40 permanent	↑ gal-3 lvl in persistent or permanent AF pts compared to paroxysmal AF pts gal-3 as an independent determinant of LAA thrombus in AF patients	gal-3 measured in serum by ELISA
[23]	2021	98 AF pts	pts divided by AF duration: 47 pts with AF ≤ 48 h and 51 pts with AF > 48 h	no difference in gal-3 lvl between the groups	gal-3 measured in plasma by ELISA
[25]	2021	51 paroxysmal AF pts with progression to persistent AF	162 paroxysmal AF pts w/o progression to persistent AF	↑ gal-3 lvl in pts with AF progression gal-3 concentration associated with AF progression	gal-3 measured in plasma by ELISA

↑ increased, *AF* atrial fibrillation,*ELISA* enzyme-linked immunosorbent assay, *gal-3* galectin-3, *h* hours, *LAA* left atrial appendage, *LAVI* left atrial volume index, *LV* left ventricular, *lvl* levels, *MS* metabolic syndrome, *NOAF* new-onset atrial fibrillation, *pts* patients, *ref.* reference, *SR* sinus rhythm, *w/o* without

### Galectin-3 in patients with atrial fibrillation undergoing cardioversion

Begg et al. examined gal-3 levels as a potential prognostic factor of AF recurrence after direct current cardioversion (DCCV). They included patients who underwent DCCV for AF and compared them to healthy controls. They demonstrated that there was no difference in gal-3 levels between these groups. Moreover, gal-3 levels did not predict AF recurrence after DCCV [26]. Consistently, Kisheva et al. examined gal-3 levels as a predictive factor of AF recurrence in patients suffering from AF after sinus rhythm restoration. It was shown that gal-3 levels did not affect the number of AF recurrences after sinus rhythm restoration [27].

A similar study was conducted by Gürses et al. They evaluated the group of 90 patients suffering from persistent AF who underwent DCCV. As presented, 28 patients experienced early AF recurrence within 3 months. Contrary to the studies above, this research group showed that the patients with AF recurrence had higher gal-3 levels compared to those who did not experience AF recurrence after the procedure [28].

Wałek et al. examined correlations between the levels of gal-3 and the echocardiographic parameters of the left atrium and left ventricle in patients suffering from persistent AF with left atrial enlargement qualified for DCCV. The authors observed negative correlations between the concentrations of gal-3 and left atrial: dimensions, volume, contractility, and compliance. Moreover, negative correlations were found between the levels of gal-3 and left ventricular: volume and contractility [29]. All studies discussed in this section with additional information have been presented in Table 3.

**Table 3:** Summary of recent research investigating the role of galectin-3 in patients with atrial fibrillation undergoing cardioversion

**Ref.**	**Year**	**Population**	**Comparison**	**Outcome**	**Methods**
[26]	2017	79 pts undergoing DCCV for AF	40 age-and-disease-matched controls	no significant difference in gal-3 lvl between the groups gal-3 lvl not predictive for AF recurrence after DCCV	gal-3 measured in serum by ELISA
[28]	2019	28 persistent AF pts undergoing DCCV with AF recurrence	62 persistent AF pts undergoing DCCV w/o AF recurrence	↑ gal-3 lvl in pts with AF recurrence	gal-3 measured in serum by ELISA
[27]	2021	67 AF pts after SR restoration	pts divided according to AF recurrence	the number of AF recurrences after SR restoration not affected by gal-3 lvl	gal-3 measured in serum by ELISA
[29]	2021	63 persistent AF pts with LA enlargement undergoing DCCV	correlations between gal-3 lvl and echocardiographic parameters	gal-3 lvl negatively correlated with LA: dimensions, volume, compliance and contractility gal-3 negatively correlated with the LV: volume and contractility	gal-3 measured in serum by ELISA

↑ increased, *AF* atrial fibrillation, *DCCV* direct current electrical cardioversion, *ELISA* enzyme-linked immunosorbent assay, *gal-3* galectin-3, *LA* left atrial, *LV* left ventricular, *lvl* levels, *pts* patients, *ref.* reference, *SR* sinus rhythm, *w/o* without

### Galectin-3 as a predictor of atrial fibrillation recurrence after ablation

Wu et al. analyzed plasma concentrations of gal-3 in patients with persistent AF without coexisting structural heart disease who underwent first-time catheter ablation and compared them to healthy controls. The levels of gal-3 were higher in patients suffering from persistent AF. Furthermore, the concentrations of gal-3 were increased in patients with recurrence of AF after ablation, compared to patients without AF recurrence. Moreover, gal-3 was an independent predictor of AF reappearance after catheter ablation [30]. Similarly, Ruan et al. analyzed the relationship between the preoperative level of gal-3 and AF recurrence in patients undergoing radiofrequency catheter ablation (RFCA). It was shown that patients who experienced the recurrence of AF after the RFCA presented higher levels of gal-3 (measured at baseline) than patients who did not develop AF again after the procedure. It was proved that gal-3 could be considered an independent predictor of AF reappearance after the procedure [31]. Consistently, Lee et al. investigated the correlation between levels of gal-3 and the risk of atrial tachyarrhythmias recurrence in patients who underwent ablation for AF. Increased levels of gal-3 were associated with an increased risk of atrial tachyarrhythmias recurrence [32].

Takemoto et al. compared the levels of gal-3 measured in intracardiac serum obtained from the coronary sinus and left atrium between patients with persistent AF and patients with paroxysmal AF. Higher levels of this biomarker were found in patients with persistent AF. They also showed that gal-3 was an independent predictor of atrial tachyarrhythmia recurrence after a single ablation procedure [33].

Clementy et al. investigated gal-3 levels and left atrial diameter (LAD) as potential predictive factors of AF recurrence in patients suffering from AF who underwent ablation. Patients who experienced AF recurrence after the procedure appeared to have higher gal-3 levels and larger left atrium. Gal-3 levels and LAD were independent predictors of AF recurrence [34].

Contrary results were obtained by Kornej et al. They conducted a study including AF patients who underwent catheter ablation and compared them to AF-free controls. It was shown that gal-3 levels were higher in AF patients compared to AF-free controls. However, the authors concluded that gal-3 did not help predict the rhythm outcome of the catheter ablation [35]. Another research team had similar findings: Celik et al. analyzed gal-3 levels as a predictive factor of AF recurrence in patients who underwent PVI. It was proven that gal-3 levels were not associated with AF recurrence. Moreover, PVI did not significantly affect serum gal-3 levels [36].

Berger et al. studied the relationship between gal-3 levels and the probability of AF recurrence in patients undergoing thoracoscopic surgery for AF. A higher recurrence rate was observed in patients with increased gal-3 levels after ablation compared to baseline than in patients with unchanged or decreased gal-3 level [37].

Yalcin et al. evaluated patients suffering from paroxysmal AF undergoing cryoballoon-based AF ablation. They studied the relationship between the levels of gal-3 and left atrium fibrosis assessed by delayed-enhancement magnetic resonance imaging (DE-MRI) and atrial electromechanical delay (AEMD). They proved that the concentrations of gal-3 were independently correlated with the extent of left atrium fibrosis detected with DE-MRI. Moreover, the levels of gal-3 correlated with intra- left and inter-AEMD [38].

Aksan et al. investigated the association between gal-3 level and low voltage areas (LVA) severity in patients suffering from paroxysmal AF who underwent PVI. It was proved that gal-3 levels were higher in paroxysmal AF patients with moderate and severe LVA compared to those with mild LVA. Moreover, gal-3 levels were higher in paroxysmal AF patients with LVA than in those without LVA [39]. All studies discussed in this section with additional information have been presented in Table 4.

**Table 4:** Summary of recent research investigating galectin-3 as a predictor of arrhythmia recurrence after ablation in patients with atrial fibrillation

**Ref.**	**Year**	**Population**	**Comparison**	**Outcome**	**Methods**
[30]	2015	50 persistent AF pts w/o coexisting structural heart disease undergoing first-time catheter ablation	46 healthy controls	↑ gal-3 lvl in AF pts compared to healthy controls ↑ gal-3 lvl in AF pts with AF recurrence compared to AF pts w/o AF recurrence gal-3 as an independent predictor of AF recurrence after ablation	gal-3 measured in plasma by the Luminex technology
[35]	2015	105 AF pts undergoing catheter ablation	14 AF-free controls	↑ gal-3 lvl in AF patients compared to AF-free controls gal-3 lvl not useful for predicting rhythm outcome of catheter ablation	gal-3 measured in plasma by ELISA
[38]	2015	33 paroxysmal AF pts undergoing cryoballoon-based AF ablation	relationship between gal-3 lvl and LA fibrosis by DE-MRI and AEMD	gal-3 lvl independently correlated with extent of LA fibrosis detected with DE-MRI gal-3 lvl correlated with intra- left and inter- AEMD	gal-3 measured in serum by ELISA
[34]	2016	55 AF pts undergoing ablation with AF recurrence	105 AF pts who underwent ablation w/o AF recurrence	↑ gal-3 lvl and larger left atrium in pts with AF recurrence gal-3 lvl and LAD as independent predictors of AF recurrence	gal-3 measured in serum by ELFA
[33]	2016	55 AF pts after ablation	pts divided by AF type	↑ gal-3 lvl in intracardiac serum in persistent AF pts compared to paroxysmal AF pts gal-3 lvl as an independent predictor of AT recurrence after a single ablation procedure	gal-3 measured in serum from CS and LA by ELISA
[37]	2018	98 AF pts undergoing thoracoscopic AF surgery	pts divided by gal-3 levels measured after procedure and compared to baseline	↑ recurrence rate in pts with ↑ gal-3 lvl after ablation compared to pts with ↓ or unchanged gal-3 lvl	gal-3 measured in serum by ELISA
[36]	2019	50 paroxysmal AF pts undergoing PVI	pts divided according to AF recurrence gal-3 levels at different timepoints (before PVI, after PVI at 6 and 12 months)	no effect of PVI on gal-3 lvl gal-3 lvl not associated with AF recurrence	gal-3 measured in serum by ELISA
[32]	2022	75 pts undergoing ablation for AF	pts divided according to AT recurrence	↑ gal-3 lvl associated with an increased risk of AT recurrence	gal-3 measured in serum by ELISA
[39]	2022	115 paroxysmal AF pts undergoing PVI	pts divided by LVA severity	↑ gal-3 lvl in paroxysmal AF pts with LVA compared to those w/o LVA ↑ gal-3 lvl in paroxysmal AF pts with moderate and severe LVA compared to those with mild LVA	gal-3 measured in serum by ELISA
[31]	2022	153 pts undergoing RFCA	pts divided according to AF recurrence	↑ gal-3 lvl at baseline in pts with AF recurrence after RFCA compared to pts w/o AF recurrence ↑ preoperative gal-3 lvl as an independent predictor of AF recurrence in pts undergoing RFCA	gal-3 measured in serum by ELISA

↓ decreased, ↑ increased, *AEMD* atrial electromechanical delay, *AF* atrial fibrillation, *AT* atrial tachyarrhythmia, *CS* coronary sinus, *DE-MRI* delayed-enhancement magnetic resonance imaging, *ELFA* Enzyme-Linked Fluorescent Assay, *ELISA* enzyme-linked immunosorbent assay, *gal-3* galectin-3, *LA* left atrial, *LAD* left atrial diameter, *LVA* low voltage area, *lvl* levels, *pts* patients, *PVI* pulmonary vein isolation, *Ref*. reference, *RFCA* Radiofrequency Catheter Ablation, *SR* sinus rhythm, *w/o* without

### Galectin-3 in patients with atrial fibrillation undergoing surgeries and other invasive procedures

Szadkowska et al. evaluated patients with first acute myocardial infarction who underwent primary percutaneous coronary intervention with stent implantation. The authors showed that new-onset AF was independently associated with increased levels of gal-3 (particularly > 16 ng/mL) [40]. Similarly, Wang et al. evaluated patients admitted with acute myocardial infarction (AMI). The authors compared patients who developed post-AMI new-onset AF to those who did not. It was evidenced that the first group had increased levels of gal-3. Furthermore, the plasma concentration of gal-3 was found as an independent predictor of post-AMI new-onset AF [41].

Richter et al. hypothesized that gal-3 level could independently predict postoperative atrial fibrillation (POAF). Based on these assumptions, they enrolled patients undergoing elective cardiac surgeries. They proved that the levels of gal-3 were higher in patients who developed POAF compared to those who did not develop it. Moreover, gal-3 levels were an independent predictor of POAF and mortality after cardiac surgery [42]. Erdem et al. obtained similar results, investigating patients undergoing coronary artery bypass graft (CABG) surgery. It was shown that gal-3 could predict POAF possessing high specificity and sensitivity [43].

Aksan et al. investigated gal-3 levels as a marker of AF recurrence expressed as atrial high-rate episodes (AHRE) in patients who underwent cardiac resynchronization therapy. The study revealed that gal-3 levels were higher in patients with AHRE than those without AHRE. Also, there was a positive correlation between gal-3 levels and the percent of time spent in total AHRE [44].

Tan et al. investigated patients suffering from heart failure (HF), dividing them based on the coexistence of AF. It was proved that gal-3 predicted HF-related hospitalization, but only in patients with coexisting AF [45]. All studies discussed in this section with additional information have been presented in Table 5.

**Table 5:** Summary of recent research investigating the role of galectin-3 in patients with atrial fibrillation undergoing surgeries and other invasive procedures

**Ref.**	**Year**	**Population**	**Comparison**	**Outcome**	**Methods**
[40]	2013	145 pts with first AMI treated with pPCI with stent implantation	pts divided by gal-3 lvl: 36 pts with the highest gal-3 lvl (4^th^ quartile, > 16 ng/mL) 109 pts with gal-3 lvl ≤ 16 ng/mL:	NOAF independently associated with gal-3 lvl > 16 ng/mL	gal-3 measured in serum by ELFA
[45]	2021	261 HF pts with AF	838 HF pts w/o AF	gal-3 predicted HF-hospitalization only in AF pts	gal-3 measured in serum by ELISA
[44]	2021	31 CRT pts with AHRE	77 CRT pts w/o AHRE	↑ gal-3 lvl in CRT pts with AHRE compared to CRT pts w/o AHRE positive correlation between gal-3 levels and a percent of time spent in total AHRE	gal-3 measured in plasma by ELISA
[42]	2022	475 pts undergoing elective cardiac surgery	pts divided into 2 groups: (i) 200 pts with POAF, and (ii) 275 pts w/o POAF	↑ gal-3 lvl in pts with POAF gal-3 as an independent predictor of POAF and mortality after cardiac surgery	gal-3 measured in serum by ELISA
[41]	2022	18 AMI pts with post-AMI NOAF	199 AMI pts w/o post-AMI NOAF	↑ gal-3 lvl in pts with post-AMI NOAF plasma gal-3 as an independent predictor of post-AMI NOAF	gal-3 measured in plasma by ELISA
[43]	2022	26 pts undergoing CABG surgery with POAF	24 pts undergoing CABG surgery w/o POAF	gal-3 lvl predictor of POAF after CABG with high specificity and sensitivity	gal-3 measured in plasma by ELISA

↑ increased, *AF* atrial fibrillation, *AHRE* atrial high-rate episode, *AMI* acute myocardial infarction, *CABG* coronary artery bypass graft, *CRT* cardiac resynchronization therapy, *ELFA* Enzyme-Linked Fluorescent Assay, *ELISA* enzyme-linked immunosorbent assay, *gal-3* galectin-3, *HF* heart failure, *lvl* levels, *NOAF* new-onset atrial fibrillation, *POAF* postoperative atrial fibrillation, *pPCI* primary percutaneous coronary intervention, *pts* patients, *Ref.* reference, *w/o* without

## Conclusions and future perspectives

Many studies regarding gal-3 as a biomarker of AF have been conducted so far. The main findings from these studies are that (i) there is a correlation between higher levels of gal-3 and activation of the profibrotic pathway, (ii) the concentration of this biomarker differs between patients depending on the type of AF (paroxysmal, persistent, and permanent), (iii) gal-3 is a promising biomarker to predict the recurrence of AF in patients after ablation and POAF after cardiac surgery.

Almost all research groups confirmed higher levels of gal-3 in patients with AF compared to non-AF controls [13–15, 18, 19]. Begg et al. obtained contrary results in 2 studies: (i) comparing AF patients undergoing ablation to healthy controls and (ii) comparing AF patients undergoing DCCV to age-and-disease-matched controls [16, 26]. The potential rationale for this observed difference may be found in the characteristics of the included population. Patients undergoing ablation are most commonly relatively young, and their AF cannot be permanent. Similarly, patients undergoing DCCV are often diagnosed quite recently, and thus the fibrosis process is less advanced than it would be after, e.g., 20 years of AF occurrence.

The next conflicting findings regard the predictive value of gal-3 for AF recurrence after DCCV. Most research found no predictive value of this biomarker in this use [26, 27]. Nevertheless, Gürses et al. showed that patients with AF recurrence after DCCV had higher levels of gal-3 [28]. Moreover, in the multivariate analysis, they found that serum gal-3 level was independently associated with early recurrence of AF after a successful DCCV. This discrepancy with other studies requires further investigation and a search for an explanation.

**Fig. 3:**
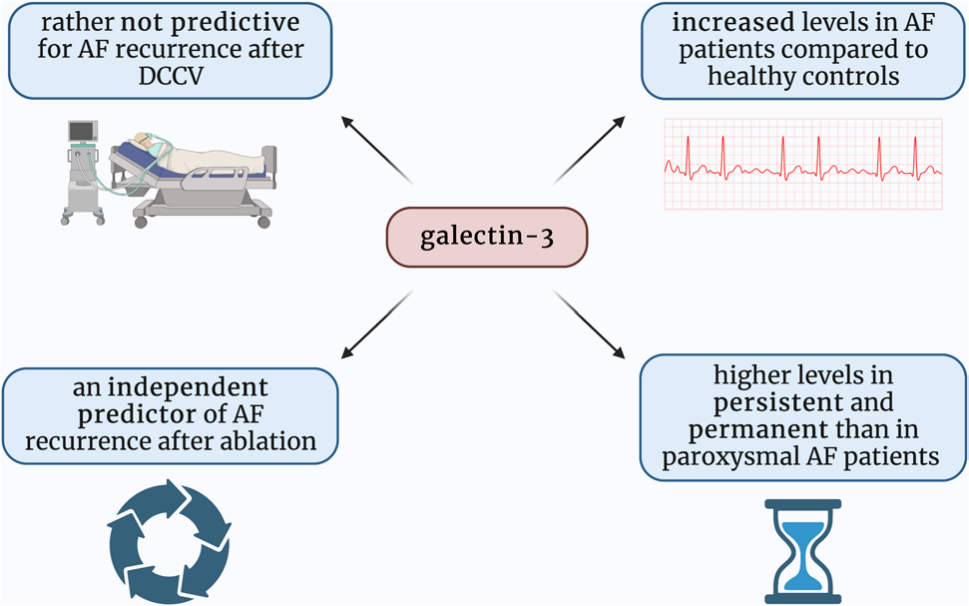
Graphical summarization of the role of galectin-3 as a potential diagnostic, prognostic, and predictive biomarker in atrial fibrillation; AF—atrial fibrillation; DCCV—direct current electrical cardioversion

Although the research considered in this manuscript addressed many aspects of the role of gal-3 in AF, most of it was conducted on small groups of patients. Therefore, more extensive research, optimally a randomized clinical trial, would be of great value to standardize and confirm the previous findings, particularly the thesis regarding the role of gal-3 as a predictive biomarker of AF recurrence after ablation (Fig. 3).

Further improvement in our knowledge about the role of gal-3 in AF would lead to a better understanding of the pathogenesis of this disease. Significantly, it could contribute to the earlier diagnosis of AF, even before the physical signs of an irregular heart rhythm. As paroxysmal AF is very difficult to spot during the electrocardiographic examination, establishing a biomarker enabling the diagnosis of AF based on its concentration would help diagnose AF at its earliest stages.

Gal-3 acts on fibroblasts’/myofibroblasts’ migration and proliferation. Macrophages are critical mediators of this process since gal-3 is most expressed in tissue-resident macrophages [46]. This plays a crucial role in activating a pro-fibrotic phenotype of macrophages and the following fibroblasts’/myofibroblasts’ activation [47]. Although much is known, there are still gaps in evidence regarding the exact role of gal-3 in the fibrosis process. Therefore, further research on the effects of gal-3 inhibition in preclinical and clinical settings may contribute to a better understanding of the gal-3 role in fibrosis and AF pathogenesis and a possible introduction of a new targeted therapy in AF. In conclusion, future studies are recommended to fully confirm the role of gal-3 as an AF biomarker and open doors for the new targeted therapy in this disease.
